# Efficacy of Pembrolizumab and Nivolumab in Crossing the Blood Brain Barrier

**DOI:** 10.7759/cureus.4446

**Published:** 2019-04-12

**Authors:** Haisam Abid, Kanramon Watthanasuntorn, Omid Shah, Rosana Gnanajothy

**Affiliations:** 1 Internal Medicine, Bassett Medical Center, Cooperstown, USA; 2 Oncology, Bassett Medical Center, Cooperstown, USA

**Keywords:** immunotherapy, blood-brain barrier, non-small cell lung cancer, pembrolizumab, nivolumab, pd-1 antibodies

## Abstract

Metastatic brain tumors are the leading cause of central nervous system malignancies in adults, surpassing primary central nervous system with non-small cell lung cancer, accounting for more than 50% of all cases. The emergence of immunotherapies such as antibodies targeting the immune check points has led to significant advancement in the field of cancer treatment since these approaches have overwhelmingly impacted outcomes in patients with metastatic non-small cell lung cancer. Here we report two cases of metastatic non-small cell lung cancer treated with immunotherapy. While one patient achieved an excellent systemic response but developed new metastatic brain lesions, the other showed remarkable systemic as well as central response. These cases highlight variable central nervous system penetration of programmed death 1 (PD-1) antibodies, and we also review the available literature on blood brain barrier permeability of PD-1 antibodies.

## Introduction

Pembrolizumab and nivolumab are humanized monoclonal antibodies that are directed against human cell surface receptor programmed death 1 (PD-1) with potential immune check point inhibitory and anti-neoplastic activities [1]. In patients with advanced non-small cell lung cancer with programmed death ligand 1 (PD-L1) expression on at least 50% of tumor cells, the estimated rate of overall survival at six months was 80.2% in the immunotherapy group versus 72.4% in the chemotherapy group [2]. According to recent researches, most cases of metastatic non-small cell lung cancer are associated with activation of PD-1. The T-cell responses to antigens are regulated by co-stimulators and co-inhibitors (the immune checkpoints). These check points are central to prevent autoimmunity and tissue damage when the immune system is working against foreign antigens [3]. Cancer cells have PDL-1 expression and bind to PD-1 on lymphocytes thus inhibiting the body to kill the cancerous cells on its own [4]. Both pembrolizumab and nivolumab inhibit PD-1 on the lymphocytes thus allowing the immune system to target and destroy cancer cells.

## Case presentation

Case 1

**Figure 1:**
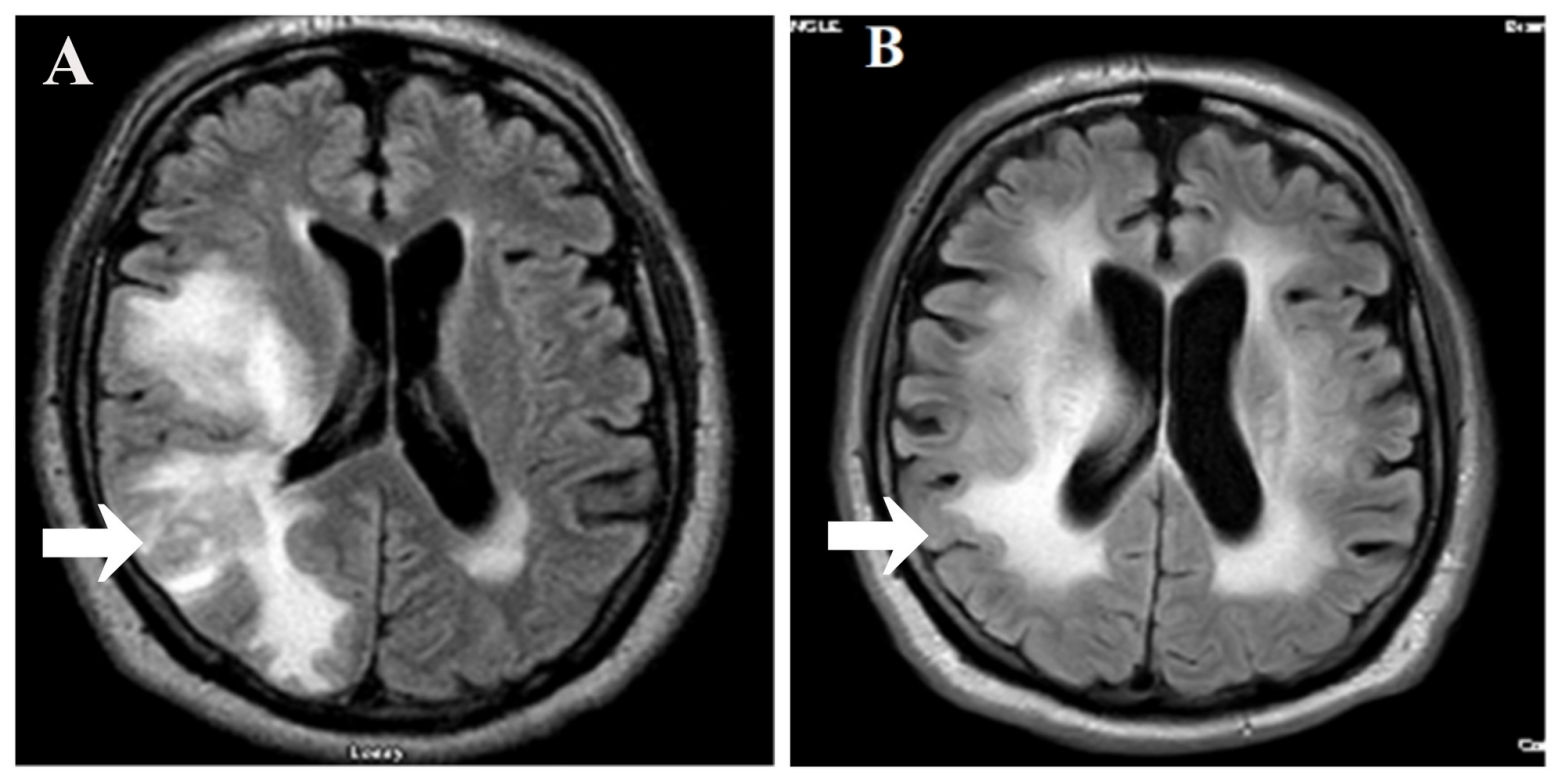
(A) Magnetic resonance imaging (MRI) of the brain with/without contrast showing new metastatic hemorrhagic lesion in the right cerebral hemisphere as compared to MRI of the brain with/without contrast (B) done after five months of treatment with pembrolizumab.

A 65-year-old male, former smoker, with a 44 pack-year smoking history presented with left upper extremity weakness and numbness that lasted for approximately half an hour. The patient denied any focal neurological deficits in other extremities. Computed tomography (CT) and magnetic resonance imaging (MRI) of the head showed intracerebral hemorrhagic metastases. CT of the chest/abdomen/pelvis showed right lower lobe lung lesions suspicious for cancer along with mediastinal and right inguinal adenopathy and a right acetabular lytic lesion. Biopsy of the right inguinal nodes showed cancer metastases morphologically and immunohistochemically identified to be adenocarcinoma, probably from lung primary as malignant cells were positive for cytokeratin AE1/AE3, thyroid transcription factor 1 (TTF-1) and cytokeratin 7 (CK7), while negative for CK20, p40, napsin, and CK5/6. The pathology report further revealed programmed death ligand 1 (PD-L1) > 100%, while epidermal growth factor receptor (EGFR), anaplastic lymphoma kinase (ALK) and receptor tyrosine kinase (ROS1) mutations were negative. The patient was first treated with radiotherapy to the brain and right hip followed by immunotherapy with pembrolizumab. Subsequent CT of the chest/abdomen/pelvis showed complete resolution of the disease and an MRI of the brain did not show any new lesions, but the patient developed forgetfulness and shuffling gait and the etiology was unclear. Initial imaging showed response in both central nervous system (CNS) and systemic disease; however, repeat imaging after five months of therapy showed control of disease outside CNS while MRI of the brain showed disease progression as patient developed new sub-ependymal metastatic lesions (Figures 1A, 1B). Biopsy was deferred as this was thought to be too invasive and the family refused it as well. Imaging findings and poor prognosis of the disease were discussed with the patient and his family, after which they decided to pursue hospice palliative care at home with no additional interventions.

Case 2

**Figure 2:**
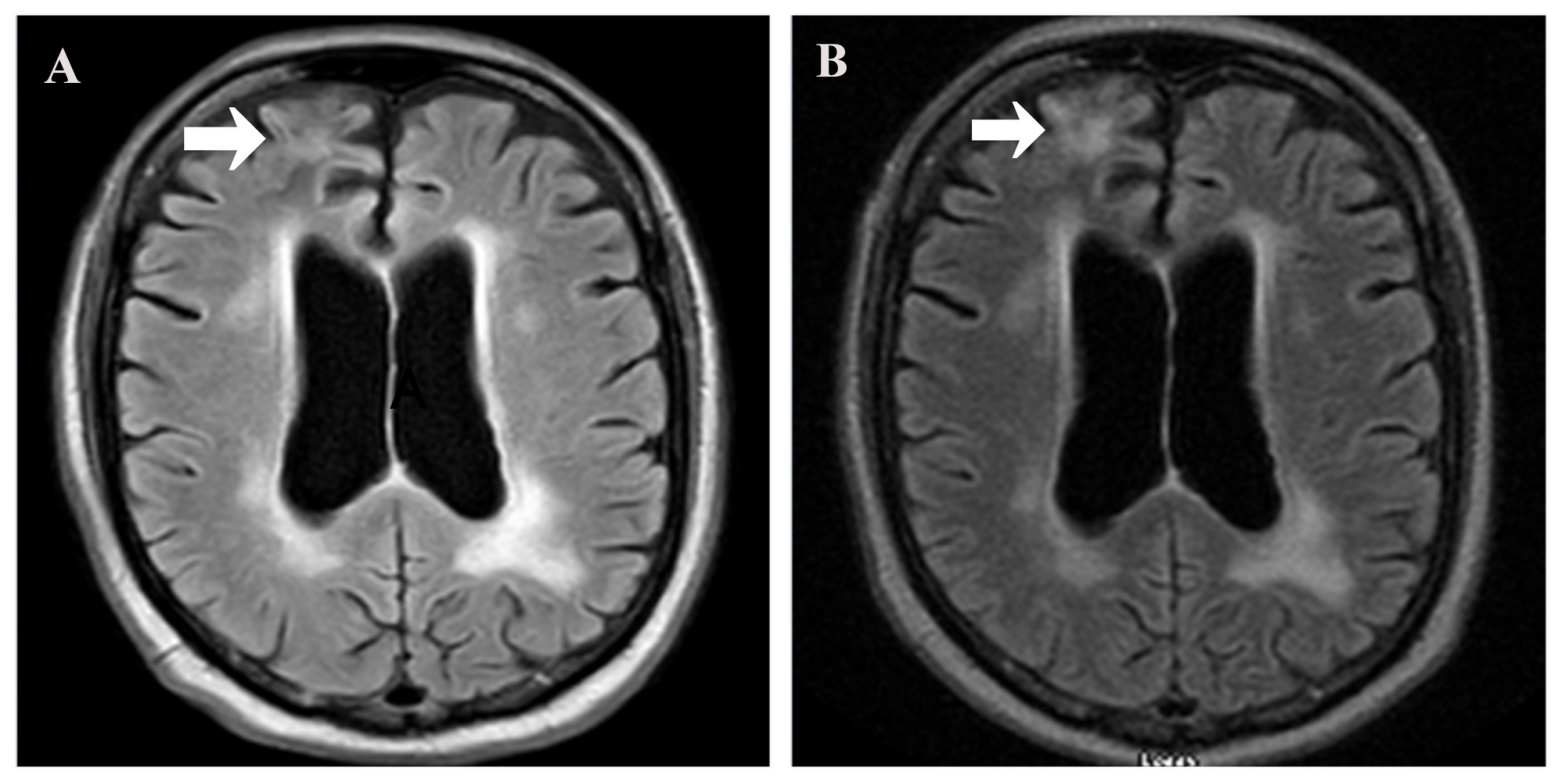
(A) Magnetic resonance imaging (MRI) of the brain with/without contrast showing disappearance of lesion in the right frontal lobe and stable lesions in other cerebral lobes as compared to MRI with/without contrast (B) done after six months of nivolumab therapy.

A 61-year-old male with a 30 pack-year smoking history came for evaluation of fatigue, significant weight loss, and poor balance with multiple falls over a period of three months. Initial workup including CT of the chest/abdomen/pelvis showed a large right paramediastinal mass with metastatic lesions involving the liver, retroperitoneal and left gluteal lymph nodes. CT of the brain was concerning for metastatic lesions of the left occipital and frontoparietal lobes. Biopsy of a left gluteal lymph node revealed cancer metastasis morphologically and immunohistochemically consistent with pulmonary adenocarcinoma as tumor cells were positive for AE1/AE3, CK7, TTF-1, and napsin-A, while negative for CK5/6, p40, melan and CK20. The tissue sample was inadequate for PD-L1 testing but EGFR, ALK, and ROS-1 mutations were absent. The patient was started on conventional chemotherapy with follow-up MRI of the brain consistent with previous CT findings. Because of poor tolerance of chemotherapy and cytopenias, therapy was changed to nivolumab based on high tumor mutation burden detected by FoundationOne testing. The patient received nivolumab, and metastatic lesions in the brain were stable and significant reduction in lung cancer and metastatic lesions outside CNS was seen on MRI of the brain (Figures 2A, 2B) and CT of the chest/abdomen/pelvis, respectively. Symptoms associated with the metastatic brain lesions disappeared completely and his general condition showed remarkable improvement.

## Discussion

Conventional therapies such as chemotherapy, radiation, and surgery, although effectual, are unsuccessful in completely eradicating residual cancerous cells in stage IV non-small cell lung cancer (NSCLC). However the emergence of checkpoint inhibitors has led to significant progress in the field of cancer therapeutics [5].

Goldberg et al. determined the efficacy and safety of pembrolizumab in 18 patients with NSCLC. Ten of these patients received prior local therapy for brain lesions but demonstrated disease progression. Typically, radiotherapy has good CNS control but it has several side effects such as decline in cognitive function or symptoms related to radiation necrosis. Brain metastasis response rate was observed to be 33% (n=6) with rare treatment-related side effects. Six patients (33%) showed disease progression in the brain, while 22% were prematurely removed from the study owing to rapid systemic progression of disease. Two patients (12%) had stable disease in the CNS. It was thus established that pembrolizumab was safe and effective for patients with NSCLC with untreated or progressive brain metastases. The findings are consistent with our second case report in which nivolumab showed good CNS response but not in the first case as that patient has advanced disease. It is worth mentioning that all patients who were previously treated with stereotactic radiosurgery or whole brain radiotherapy did not have a CNS response [6]. However, according to O’Kane and Leighl, combination treatment comprising of immunotherapy and radiotherapy may show better clinical outcomes as compared to immunotherapy alone [7]. We used this dual treatment strategy for our first case but unfortunately due to his advanced disease, he did not have good outcome.

Another retrospective study conducted from 2011-2017 found that in 94 patients with brain metastasis from NSCLC who underwent immunotherapy combined other treatments (such as whole brain radiation therapy, surgery, stereotactic radiosurgery or systemic chemotherapy) with 48.3% of overall survival and 21% of median of progression-free survival. From the study, it was concluded that immune checkpoint inhibitors are efficacious in the treatment of brain metastasis [8]. Another study investigating the prospects of nivolumab on brain metastasis in five NSCLC patients, who were asymptomatic and did not receive any prior local therapy, showed one complete and one partial response with no significant neurotoxicity. It was concluded that nivolumab might have intracranial activity and no treatment-related adverse effects, as observed in our second case report [9].

## Conclusions

Immune checkpoint inhibitors might be beneficial in stage IV NSCLC patients with intracranial metastases suggestive of blood brain barrier permeability of PD-L1 inhibitors. Immunotherapy has proved to be effective in a small number of studies and requires larger studies for further evaluation. There is also discrepancy in the role of combination therapy consisting of immune modulators and radiotherapy in the treatment of brain metastasis from NSCLC and it requires further attention.
